# Comparison of laparoscopic and open inguinal–hernia repair in elderly patients: the experience of two comprehensive medical centers over 10 years

**DOI:** 10.1007/s10029-024-03004-0

**Published:** 2024-04-04

**Authors:** S. Xi, Z. Chen, Q. Lu, C. Liu, L. Xu, C. Lu, R. Cheng

**Affiliations:** 1 Department of Comprehensive Surgery, The Second Medical Center and National Clinical Research Center for Geriatric Diseases, Chinese PLA General Hospital, No. 28 Fu Xing Road, Beijing, 100853 China; 2 Department of General Surgery, First Medical Center of Chinese PLA General Hospital, No. 28 Fu Xing Road, Beijing, 100853 China

**Keywords:** Inguinal hernia, Laparoscopy, Elderly, Co-management, Complication

## Abstract

**Purpose:**

The safety of laparoscopic inguinal–hernia repair must be carefully evaluated in elderly patients. Very little is known regarding the safety of the laparoscopic approach in elderly patients under surgical and medical co-management (SMC). Therefore, this study evaluated the safety of the laparoscopic approach in elderly patients, especially patients with multiple comorbidities under SMC.

**Methods:**

From January 2012 to December 2021, patients aged ≥ 65 years who underwent open or laparoscopic inguinal–hernia repair during hospitalization were consecutively enrolled. Postoperative outcomes included major and minor operation-related complications, and other adverse events. To reduce potential selection bias, propensity score matching was performed between open and laparoscopic groups based on patients’ demographics and comorbidities.

**Results:**

A total of 447 elderly patients who underwent inguinal–hernia repair were enrolled, with 408 (91.3%) underwent open and 39 (8.7%) laparoscopic surgery. All postoperative outcomes were comparable between open and laparoscopic groups after 1:1 propensity score matching (all *p* > 0.05). Moreover, compared to the traditional care group (*n* = 360), a higher proportion of the SMC group (*n* = 87) was treated via the laparoscopic approach (18.4% vs. 6.4%, *p* = 0.00). In the laparoscopic approach subgroup (*n* = 39), patients in the SMC group (*n* = 16) were older with multiple comorbidities but were at higher risks of only minor operation-related complications, compared to those in the traditional care group.

**Conclusions:**

Laparoscopic inguinal–hernia repair surgery is safe for elderly patients, especially those with multiple comorbidities under SMC.

## Introduction

Given the advances in healthcare and medical technology, the proportions of elderly populations continue to increase. It is estimated that by 2050, there will be 382 million people over the age of 65 years in China, 31.03% of the total population. One multicenter, Chinese epidemiological study found that the prevalence of inguinal–hernia was 1.15% in people aged over 60 years [1]. The incidence of inguinal–hernia increases with advancing age given the progressive loss of tissue strength and the development of diseases that increase intra-abdominal pressure [2]. Patients aged 75 years or older constitute over 30% of all patients with inguinal–hernias in China. Inguinal–hernia repair is today one of the most common general surgeries [2]. Given their multiple comorbidities and frailty, elderly inguinal–hernia patients are at higher risks of perioperative mortality and complications than others.

To ensure that invasive treatment is effective and to reduce perioperative complications, the best operative approach depends on the patient’s physical condition. The traditional open approach with the patient under local or general anesthesia is the mainstream treatment for elderly inguinal–hernia patients. However, postoperative pain and infection are common and hospital stays longer in the elderly [3]. In recent years, the safety and efficacy of laparoscopic inguinal–hernia repair have been well-established in general populations [3, 4]. Although the incidences of postoperative urine retention and seroma are higher than after the traditional treatment, the laparoscopic approach is associated with less postoperative pain and more rapid recovery in general populations [4–7]. However, patients must be placed under general anesthesia. Several studies have shown that the laparoscopic approach is also appropriate for elderly patients of ASA classes I–II; the incidences of mortality and complications remain low [5, 8, 9]. However, the safety of laparoscopic inguinal–hernia repair has not been sufficiently established for elderly Chinese patients.

In addition, the traditional model may not provide adequate perioperative care and optimal support that prevents and manages postoperative complications in elderly surgical patients [10]. The collaborative surgical and medical co-management (SMC) model [11] replaces a simple reactive consultation with continuing pro-active service, and may thus optimize the quality of perioperative care, improving survival and post-operation outcomes. SMC improved clinical outcomes and reduced postoperative complications [11–13]. To date, very little is known regarding the clinical safety of laparoscopic inguinal–hernia repair in elderly patients under SMC. Therefore, the present study compares the postoperative outcomes of open and laparoscopic inguinal–hernia repair in elderly patients, in particular patients under SMC. We hypothesized that the laparoscopic approach would be as safe as the open approach, especially in patients under SMC.

## Materials and methods

### Study population and treatments

This was a two-center, retrospective cohort study. Participants were consecutively recruited from the First and Second Medical Centers of Chinese PLA General Hospital, Beijing, China, and underwent open or laparoscopic inguinal–hernia repair during hospitalization between January 2012 and December 2021. Patients aged 65 years or older undergoing emergency or elective inguinal–hernia repair were eligible. The diagnosis and the use of open or laparoscopic inguinal–hernia repair were confirmed by examining the operative notes.

### Traditional care

In the First Medical Center of Chinese PLA General Hospital, all included patients were treated by general surgeons who delivered traditional care. The anesthesiologist performed an assessment before surgery. Specialists were consulted if co-existing medical conditions might affect the surgical outcomes. Open or laparoscopic inguinal–hernia repair was performed by senior surgeons after considering all opinions of the general surgeon, anesthesiologist, specialists, and the patients themselves.

### Surgical and medical co-management (SMC)

**Fig. 1:**
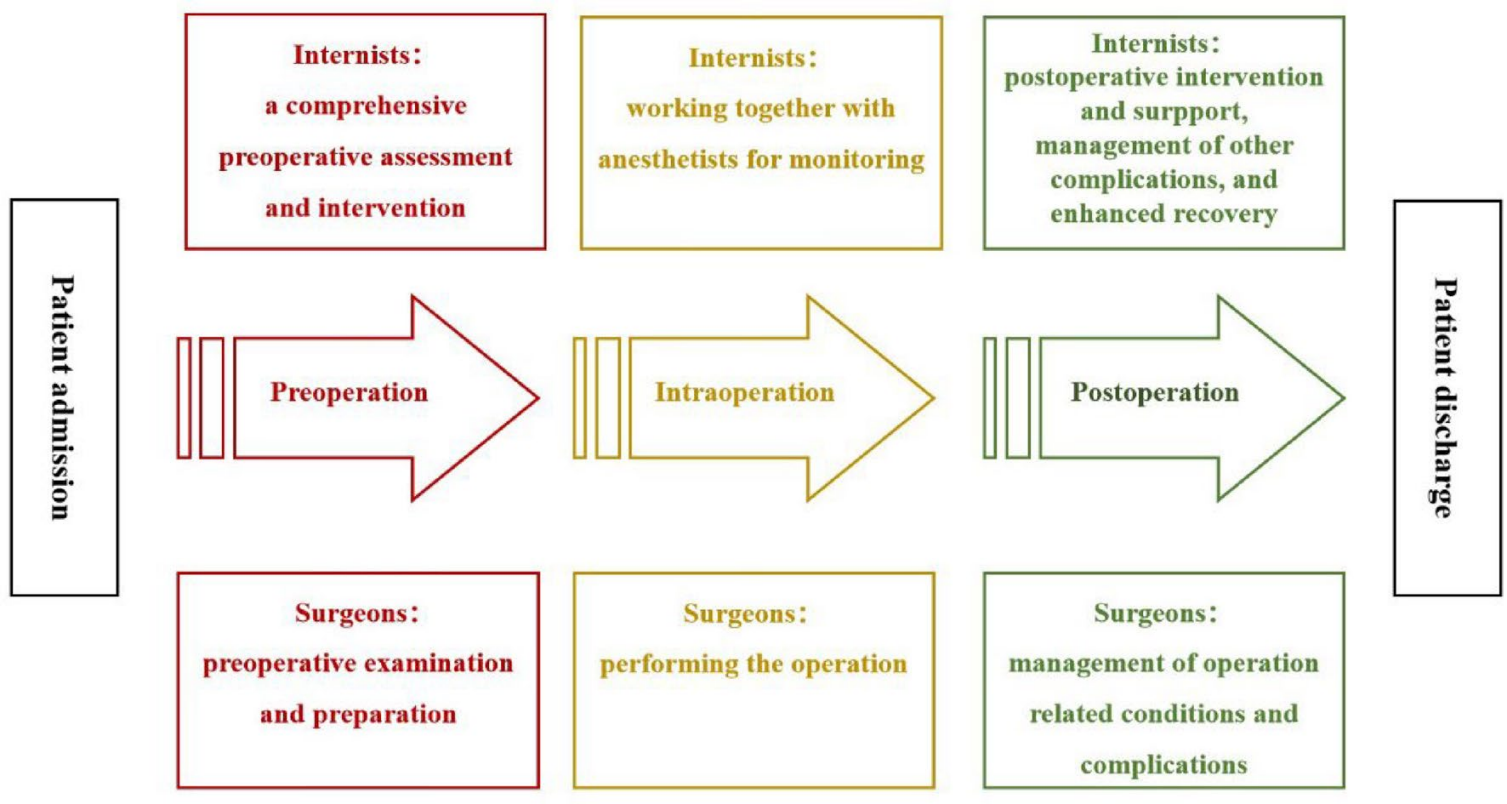
Flow diagram of surgical and medical co-management (SMC)

In the Second Medical Center of Chinese PLA General Hospital, all included patients were treated by the perioperative management SMC team, of which the general surgeons were also from the First Medical Center. SMC emphasizes patient-centered care, seeks to reduce perioperative comorbidity and mortality, and aims to improve clinical outcomes and quality of life. As Fig. 1 shows, SMC is initiated when surgical intervention is indicated. A comprehensive pre-operative evaluation is performed by internists, surgeons, anesthetists, and other experts (cardiologists and neurologists). After thorough evaluation, appropriate preoperative interventions are conducted by internists, focusing on correction of modifiable risk factors, consultations with specialists, optimization of medication, and management of any required antithrombotic therapy. After comprehensive pre-operative evaluation and intervention, the surgical approach and anesthesia method are selected via discussion. The internists work with the anesthetists to ensure that the vital signs remain stable during operation. Postoperatively, the management team implements interventions that seek to reduce the risks of operation-related complications, infection, delirium, cardiopulmonary complications, and deep venous thrombosis. To enhance recovery, the focus is on adequate pain management, nutrition support, and general postoperative care. Moreover, we assist patients to become mobile early and guide them through rehabilitation exercises.

### Ethics statement

The study protocol was approved by the Clinical Research Ethics Committee of the Chinese PLA General Hospital (approval no. S2022-664-01) and was conducted in accordance with the Declaration of Helsinki. Written informed consent from patients was not required because of the retrospective nature of the study.

### Data collection

The clinical, electronic medical records yielded patient baseline demographics, concomitant diseases, American Society of Anesthesiologists (ASA) classes, laboratory parameters, anesthesia methods, the surgical approaches, intraoperative parameters, and complications during hospitalization. Frailty was assessed using the Frailty Assessment Scale for Elderly Inguinal Hernia Patients.

### Postoperative outcomes

Postoperative outcomes included major and minor operation-related complications and other adverse events. Major operation-related complications were defined as deep incisional surgical site or organ space surgical site infections, a poked hole hernia, intestinal injury, and mechanical intestinal obstruction. Minor operation-related complications included seroma, active bleeding, hematoma of scrotum, fever, surgical site infection, urine retention, and urinary infection.

Other adverse events included delirium, venous thromboembolism (VTE), anemia, pneumonia, hepatic or renal insufficiency, coma, stroke or a cardiovascular accident (myocardial infarction or cardiac arrest requiring cardiopulmonary resuscitation), sepsis, septic shock, and death.

### Statistical analysis

Categorical variables are described as numbers with percentages (*n*, %). Univariate analyses were performed using the Chi-squared test or Fisher’s exact test for categorical variables, as appropriate. Data rankings were calculated with the aid of the rank-sum test. Continuous variables are presented as means ± standard deviations (SDs) and were compared using Student’s *t* test. Propensity scores were created for the odds of receiving laparoscopic surgery based on age, sex, body mass index (BMI), ASA class, frailty classification, coronary heart disease, hypertension, diabetes, chronic renal insufficiency, COPD, and cerebrovascular disease. Patients were matched 1:1 based on the difference in the logit of the propensity scores, with a caliper width equal to 0.2 of the logit of propensity scores standard deviation. Postoperative outcomes were compared after propensity score matching. A *p* value < 0.05 was considered statistically significant. All data analyses were conducted with the aid of SPSS Statistics software version 25.0 (IBM Corp., Armonk, New York, United States).

## Results

### Comparison of the open and laparoscopic surgery groups

A total of 447 elderly patients who underwent inguinal–hernia repair were enrolled, of whom 87 (19.5%) were under SMC. Of all patients, 408 (91.3%) underwent open and 39 (8.7%) laparoscopic surgery. The baseline clinical characteristics of the patients are listed in Table 1. There was no significant difference in any of age, sex, BMI, frailty classification, concomitant disease status, or emergency operation between the open and laparoscopic groups (all *p* ≥ 0.05) and the proportions of ASA class ≥ 3 were similar in the two groups (26.2% vs. 30.8%, *p* = 0.54). Compared to the laparoscopic group, only 72/408 (17.6%) of the open group underwent general anesthesia; the difference was significant (100% vs. 17.6%, *p* = 0.00).

**Table 1:** Clinical data of elderly patients undergoing open and laparoscopic inguinal–hernia repair

Characteristics	Unmatched			Matched			Open surgery under general anesthesia (*n* = 72)	Laparoscopic surgery (*n* = 39)	*p* value
	Open surgery (*n* = 408)	Laparoscopic surgery (*n* = 39)	*p* value	Open surgery (*n* = 39)	Laparoscopic surgery (*n* = 39)	*p* value			
Age, mean ± SD, years	75.2 ± 6.5	74.5 ± 8.0	0.58	74.2 ± 7.2	74.5 ± 8.0	0.85	75.6 ± 7.3	74.5 ± 8.0	0.47
> 75 years	188 (46.1%)	16 (41.0%)	0.55	16 (41.0%)	16 (41.0%)	1.00	36 (50.0%)	16 (41.0%)	0.37
Male, *n* (%)	395 (96.8%)	39 (100%)	0.53	39 (100%)	39 (100%)	1.00	65 (90.3%)	39 (100%)	0.09
BMI, mean ± SD, kg/m^2^	23.4 ± 2.8	23.9 ± 2.6	0.29	23.1 ± 2.4	23.9 ± 2.6	0.14	24.0 ± 4.1	23.9 ± 2.6	0.88
Coronary heart disease, *n* (%)	63 (15.4%)	7 (17.9%)	0.68	2 (5.1%)	7 (17.9%)	0.15	14 (19.4%)	7 (17.9%)	0.85
Hypertension, *n* (%)	160 (39.2%)	19 (48.7%)	0.25	13 (33.3%)	19 (48.7%)	0.17	24 (33.3%)	19 (48.7%)	0.11
Diabetes, *n* (%)	63 (15.4%)	6 (15.4%)	0.99	9 (23.1%)	6 (15.4%)	0.39	7 (9.7%)	6 (15.4%)	0.56
Chronic renal insufficiency, *n* (%)	6 (1.5%)	1 (2.6%)	1.00	1 (2.6%)	1 (2.6%)	1.00	1 (1.4%)	1 (2.6%)	1.00
COPD, *n* (%)	7 (1.7%)	1(2.6%)	1.00	1 (2.6%)	1 (2.6%)	1.00	1 (1.4%)	1(2.6%)	1.00
Cerebrovascular disease, *n* (%)	11 (2.7%)	3 (7.7%)	0.22	3 (7.7%)	3 (7.7%)	1.00	1 (1.4%)	3 (7.7%)	0.12
ASA class ≥ 3, *n* (%)	107 (26.2%)	12 (30.8%)	0.54	12 (30.8%)	12 (30.8%)	1.00	23 (31.9%)	12 (30.8%)	0.90
Frailty Scale, *n* (%)									
Normal (≥ 12)	349 (85.5%)	34 (87.2%)	0.23	37 (94.9%)	34 (87.2%)	0.51	62 (86.1%)	34 (87.2%)	0.95
Mild (8–11)	51 (12.5%)	3 (7.7%)		2 (5.1%)	3 (7.7%)		5 (6.9%)	3 (7.7%)	
Moderate (4–7)	5 (1.2%)	2 (5.1%)		0 (0.0%)	2 (5.1%)		3 (4.2%)	2 (5.1%)	
Severe (0–3)	3 (0.7%)	0 (0.0%)		0 (0.0%)	0 (0.0%)		2 (2.8%)	0 (0.0%)	
Preoperative laboratory data									
Hemoglobin, mean ± SD, g/dL	137.7 ± 14.2	139.2 ± 14.1	0.55	138.0 ± 11.7	139.2 ± 14.1	0.70	138.2 ± 14.5	139.2 ± 14.1	0.75
WBCs, (mean ± SD) × 10^9^/L	5.9 ± 2.0	5.7 ± 1.9	0.60	6.1 ± 1.9	5.7 ± 1.9	0.34	6.5 ± 3.1	5.7 ± 1.9	0.13
Platelets, (mean ± SD) × 10^9^/L	186.0 ± 54.3	171.6 ± 41.4	0.05	189.4 ± 54.2	171.6 ± 41.4	0.11	190.0 ± 57.6	171.6 ± 41.4	0.06
Total serum protein, mean ± SD, g/L	68.7 ± 34.3	64.8 ± 4.8	0.48	66.5 ± 6.1	64.8 ± 4.8	0.17	72.1 ± 35.8	64.8 ± 4.8	0.21
Serum albumin, mean ± SD, g/L	41.1 ± 3.8	40.1 ± 3.3	0.14	40.6 ± 3.8	40.1 ± 3.3	0.60	40.5 ± 4.0	40.1 ± 3.3	0.60
eGFR, mean ± SD, mL/min	69.2 ± 43.1	71.6 ± 20.1	0.73	70.4 ± 16.8	71.6 ± 20.1	0.79	70.0 ± 21.1	71.6 ± 20.1	0.69
< 60 mL/min	147 (36.8%)	10 (26.3%)	0.20	9 (23.1%)	10 (26.3%)	0.74	28 (38.9%)	10 (26.3%)	0.19
ALT, mean ± SD, U/L	17.0 ± 13.8	16.8 ± 11.4	0.94	17.1 ± 14.6	16.8 ± 11.4	0.92	15.6 ± 7.0	16.8 ± 11.4	0.48
AST, mean ± SD, U/L	19.1 ± 8.0	18.7 ± 8.2	0.74	19.1 ± 10.2	18.7 ± 8.2	0.82	18.6 ± 5.4	18.7 ± 8.2	0.98
General anesthesia, *n* (%)	72 (17.6%)	39 (100.0%)	0.00	9 (23.1%)	39 (100.0%)	0.00	72 (100.0%)	39 (100.0%)	1.00
Emergency operation, *n* (%)	28 (6.9%)	3 (7.7%)	1.00	1 (2.6%)	3 (7.7%)	0.62	16 (22.2%)	3 (7.7%)	0.05

*BMI* body mass index, *COPD* chronic obstructive pulmonary disease, *ASA* American Society of Anesthesiologists, *WBC* white blood cell, *eGFR* estimated glomerular filtration rate, *ALT* alanine aminotransferase, *AST* aspartate aminotransferase, *NSAIDs* nonsteroidal anti-inflammatory drugs, *PCA* patient-controlled analgesia

The postoperative outcomes are listed in Table 2. The laparoscopic group exhibited higher incidences of seroma (7.7% vs. 1.5%, *p* = 0.04) and hematoma of scrotum (10.3% vs. 1.7%, *p* = 0.01) compared to the open group. The postoperative fever incidence was higher in the open than the laparoscopic group (14.0% vs. 2.6%, *p* = 0.04). Whereas, under general anesthesia subgroup, all postoperative outcomes were comparable between open (*n* = 72) and laparoscopic surgery groups (*n* = 39) (all *p* > 0.05).

**Table 2:** Postoperative outcomes of elderly patients undergoing open and laparoscopic inguinal–hernia repair

Postoperative complications	Unmatched			Matched			Open surgery under general anesthesia (*n* = 72)	Laparoscopic surgery (*n* = 39)	*p* value
	Open surgery (*n* = 408)	Laparoscopic surgery (*n* = 39)	*p* value	Open surgery (*n* = 39)	Laparoscopic surgery (*n* = 39)	*p* value			
Operation-related complications	70 (17.2%)	6 (15.4%)	0.78	6 (15.4%)	6 (15.4%)	1.00	13 (18.1%)	6 (15.4%)	0.72
Major complications	0 (0.0%)	0 (0.0%)	1.00	0 (0.0%)	0 (0.0%)	1.00	0 (0.0%)	0 (0.0%)	1.00
Seroma	6 (1.5%)	3 (7.7%)	0.04	1 (2.6%)	3 (7.7%)	0.62	2 (2.8%)	3 (7.7%)	0.34
Active bleeding	2 (0.5%)	0 (0.0%)	1.00	0 (0.0%)	0 (0.0%)	1.00	0 (0.0%)	0 (0.0%)	1.00
Hematoma of scrotum	7 (1.7%)	4 (10.3%)	0.01	2 (5.1%)	4 (10.3%)	0.68	3 (4.2%)	4 (10.3%)	0.24
Postoperative fever	57 (14.0%)	1 (2.6%)	0.04	3 (7.7%)	1 (2.6%)	0.62	11 (15.3%)	1 (2.6%)	0.08
Wound infection	2 (0.5%)	0 (0.0%)	1.00	0 (0.0%)	0 (0.0%)	1.00	0 (0.0%)	0 (0.0%)	1.00
Urine retention	3 (0.7%)	0 (0.0%)	1.00	1 (2.6%)	0 (0.0%)	1.00	1 (1.4%)	0 (0.0%)	1.00
Urinary infection	1 (0.2%)	0 (0.0%)	1.00	0 (0.0%)	0 (0.0%)	1.00	0 (0.0%)	0 (0.0%)	1.00
Delirium	2 (0.5%)	0 (0.0%)	1.00	0 (0.0%)	0 (0.0%)	1.00	0 (0.0%)	0 (0.0%)	1.00
Other adverse events	6 (1.5%)	2 (5.1%)	0.31	1 (2.6%)	2 (5.1%)	1.00	0 (0.0%)	2 (5.1%)	0.12

In addition, propensity score matching was performed between open and laparoscopic groups based on patients’ demographics and comorbidities. We also found that patients undergoing open surgery were less likely to have general anesthesia (23.1% vs. 100%, *p* = 0.00). However, the difference of postoperative outcomes disappeared after propensity score matching. All postoperative outcomes were also comparable between open and laparoscopic surgery groups (all *p* > 0.05).

### A comparison of traditional care and SMC

**Fig. 2:**
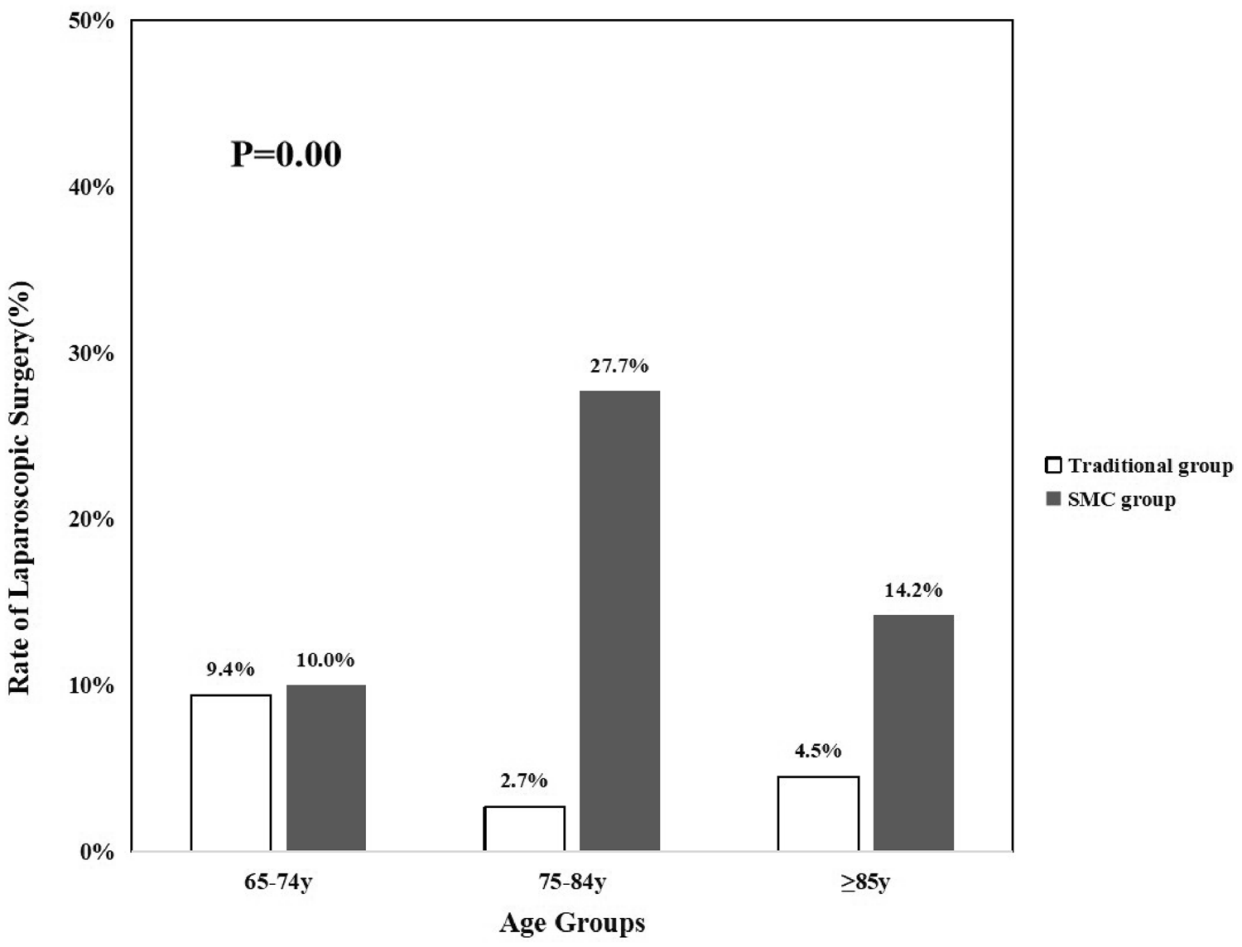
Laparoscopic surgery rates for patients of different ages in the traditional care and SMC groups. The *p* value is that for the comparison of patients of all ages in the two groups

**Fig. 3:**
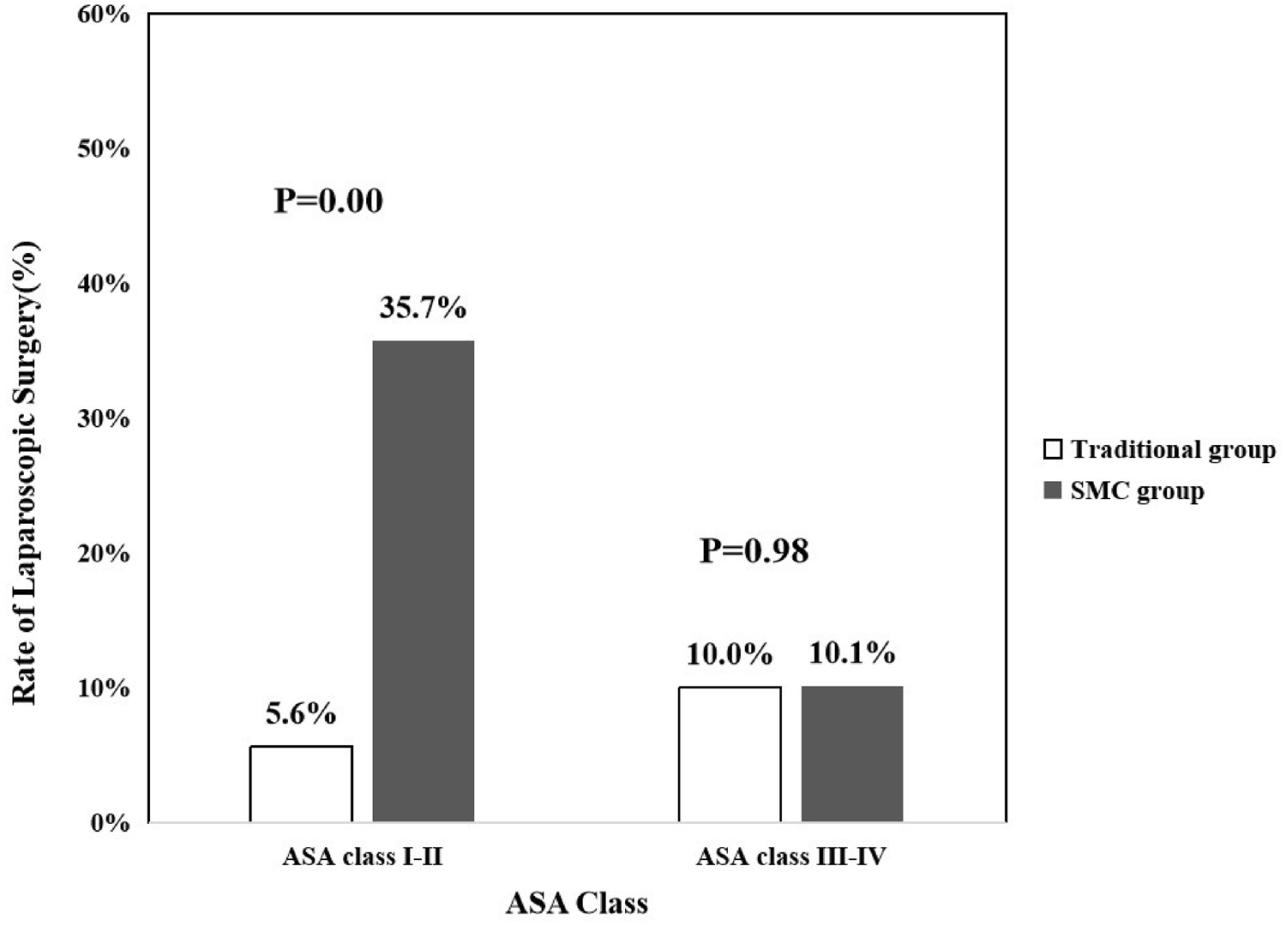
Laparoscopic surgery rates by the different ASA classes of patients in the traditional care and SMC groups

Compared to the traditional care group, the proportion of laparoscopic surgery was higher in the SMC group (6.4% vs. 18.4%, *p* = 0.00) and the proportions of laparoscopic surgery by age also differed significantly between the two groups (Fig. 2). That proportion was higher in patients aged 75 years or older under SMC (*p* = 0.00). The proportions of laparoscopic surgery by the various ASA classes also differed significantly between the two groups (Fig. 3). The proportion was higher among ASA class I–II patients under SMC (*p* = 0.00).

Thirty-nine elderly patients underwent laparoscopic surgery, with 32 cases (82.1%) undergoing the surgery in recent 2 years. Totally, there were 16 (41.0%) patients under SMC and 23 (59.0%) under traditional care. Their baseline clinical characteristics and postoperative outcomes are listed in Table 3. In terms of the clinical characteristics, patients with SMC were older, more often exhibited lower hemoglobin levels, and more commonly had hypertension or diabetes than others. The proportion of ASA class ≥ 3 patients was somewhat higher in those under SMC, but statistical significance was not attained (37.5% vs. 26.1%, *p* = 0.68). In terms of postoperative outcomes, there was no major operation-related complication in either group. Compared to patients under traditional care, patients under SMC evidenced a higher incidence of minor operation-related complications (0.0% vs. 37.5%, *p* = 0.00), principally hematoma of scrotum (0.0% vs. 25.0%, *p* = 0.02). There was no significant difference in the incidences of delirium or other adverse events between the two groups (both* p* ≥ 0.05).

**Table 3:** Clinical data and postoperative outcomes of subgroup patients under SMC and traditional care undergoing laparoscopic surgery

	SMC group (*n* = 16)	Traditional group (*n* = 23)	*p* value
Age, mean ± SD, years	79.2 ± 8.6	71.3 ± 5.9	0.00
> 75 years	13 (81.3%)	3 (13.0%)	0.00
Male, *n *(%)	16 (100.0%)	23 (100%)	1.00
BMI, mean ± SD, kg/m^2^	24.1 ± 1.8	23.8 ± 3.1	0.75
Coronary heart disease, *n* (%)	5 (31.3%)	2 (8.7%)	0.10
Hypertension, *n* (%)	12 (75.0%)	7 (30.4%)	0.01
Diabetes, *n* (%)	5 (31.3%)	1 (4.3%)	0.03
Chronic renal insufficiency, *n* (%)	1 (6.3%)	0 (0.0%)	0.41
COPD, *n* (%)	0 (0.0%)	1 (4.3%)	1.00
Cerebrovascular disease, *n* (%)	1 (6.3%)	2 (8.7%)	1.00
ASA class ≥ 3, *n* (%)	6 (37.5%)	6 (26.1%)	0.68
Frailty scale, *n* (%)			
Normal (≥ 12)	13 (81.3%)	21 (91.3%)	0.27
Mild (8–11)	1 (6.3%)	2 (8.7%)	
Moderate (4–7)	2 (12.5%)	0 (0.0%)	
Severe (0–3)	0 (0.0%)	0 (0.0%)	
Preoperative laboratory values			
Hemoglobin, mean ± SD, g/dL	133.3 ± 15.8	143.5 ± 11.3	0.03
WBCs, (mean ± SD) × 10^9^/L	5.8 ± 2.1	5.6 ± 1.7	0.76
Platelets, (mean ± SD) × 10^9^/L	173.4 ± 42.6	170.4 ± 41.5	0.83
Total serum protein, mean ± SD, g/L	64.2 ± 3.4	65.3 ± 5.6	0.50
Serum albumin, mean ± SD, g/L	39.7 ± 3.4	40.5 ± 3.2	0.48
eGFR, mean ± SD, mL/min	67.6 ± 22.9	74.5 ± 17.8	0.31
< 60 mL/min	5 (31.3%)	5 (22.7%)	0.83
ALT, mean ± SD, U/L	16.6 ± 11.3	17.0 ± 11.8	0.92
AST, mean ± SD, U/L	18.9 ± 8.9	18.5 ± 7.9	0.91
Emergency operation, *n* (%)	2 (12.5%)	1(4.3%)	0.56
Operation-related complications	6 (37.5%)	0 (0.0%)	0.00
Major complications	0 (0.0%)	0 (0.0%)	1.00
Seroma	3 (18.8%)	0 (0.0%)	0.06
Active bleeding	0 (0.0%)	0 (0.0%)	1.00
Hematoma of the scrotum	4 (25.0%)	0 (0.0%)	0.02
Postoperative fever	1 (6.3%)	0 (0.0%)	0.41
Wound infection	0 (0.0%)	0 (0.0%)	1.00
Urine retention	0 (0.0%)	0 (0.0%)	1.00
Urinary infection	0 (0.0%)	0 (0.0%)	1.00
Delirium	0 (0.0%)	0 (0.0%)	1.00
Other adverse events	2 (12.5%)	0 (0.0%)	0.16

*BMI* body mass index, *COPD* chronic obstructive pulmonary disease, *ASA* American Society of Anesthesiologists, *WBC* white blood cell, *eGFR* estimated glomerular filtration rate, *ALT* alanine aminotransferase, *AST* aspartate aminotransferase, *NSAIDs* nonsteroidal anti-inflammatory drugs, *PCA* patient-controlled analgesia

## Discussion

Laparoscopic surgery was as safe as open surgery in elderly inguinal–hernia patients, without significant difference of postoperative outcomes after propensity score matching or under general anesthesia subgroup. For patients under SMC, laparoscopic surgery was appropriate for those with multiple comorbidities associated with higher risks of minor operation-related complications.

Our results are similar to those of previous studies [5, 14–18]. We confirmed that laparoscopic surgery is as safe as open surgery for elderly inguinal–hernia patients. Compared to open surgery, laparoscopic surgery was more likely associated with seroma and hematoma of scrotum and less likely with postoperative fever [3, 19], whereas without significant difference after propensity score matching.

Our study is the first to compare the outcomes of traditional care and SMC in elderly patients undergoing laparoscopic inguinal–hernia repair. Our findings expand the results of prior works. We found that, under SMC, older patients with multiple comorbidities, associated with higher incidences of frailty and ASA class ≥ 3 status, were safely treated via laparoscopic inguinal–hernia repair. The comprehensive perioperative assessment and interventions improved the clinical outcomes and reduced postoperative complications; the mortality rates were low for patients of all ages undergoing elective hernia repair surgery [2, 14]. Compared to the traditional care group, the proportion of laparoscopic surgery was higher in the SMC group (6.4% vs. 18.4%, *p* = 0.00) perhaps because the latter surgery must be performed with patients under general anesthesia. However, elderly patients are at more risk of cognitive decline after general anesthesia than others [14, 20]. Thus, open surgery under local anesthesia accounted for 82.4% of all open surgeries in our study, associated with a shorter operative time, less postoperative pain, and lower rates of metabolic acidosis and cardiopulmonary complications [3, 14]. Therefore, although laparoscopic surgery was associated with more rapid mobilization and earlier hospital discharge, surgeons were more likely to choose open surgery when patients were under traditional care. Under SMC, internists aided surgeons to manage the entire perioperative period, and the proportion of laparoscopic surgery was thus higher to enhance recovery after surgery, as recommended by the guidelines [3]. The proportion of laparoscopic surgery was also higher among patients of ASA class of I–II under SMC, in line with the guidelines that favor expert consensus over chronological age [3, 21].

In brief, there is no uniform “single correct” surgical method for inguinal–hernia repair in elderly patients. The choice should be based on surgeon expertise and comprehensive assessment of patient- and hernia-related factors, including cognitive, functional, nutritional, socioeconomic, and affective status. International guidelines for groin hernia management [3] suggest that, when planning elective inguinal–hernia repair, medical comorbidities are the primary causes of death and must be carefully considered, especially in elderly patients. Recent studies [14, 21–23] have shown that frailty, rather than chronological age, is more prognostic of postoperative complications and mortality. Therefore, when selecting candidates for elective inguinal–hernia repair, we recommend the following. First, a comprehensive assessment should be performed after admission, with evaluation of the perioperative cardiac, pulmonary, renal, hepatic, neurological, VTE, malnutrition, anemia, delirium, and cognitive decline risks. ASA class and the Frailty score must be noted. Second, patients should be optimized in terms of modifiable risk factors, thus smoking, hypertension, diabetes, anemia, malnutrition, and frailty. Ideally, patients should quit smoking at least 6 weeks before elective repair surgery. The HgbA1c level should be controlled to below 7% in diabetics. Blood pressure should be controlled to within the target range in patients with hypertension. The hemoglobin level should exceed 10 g/L in anemic patients. Nutritional support and prehabilitation improve the nutritional condition and frailty score. Third, laparoscopic surgery is recommended in bilateral inguinal hernias for patients who can tolerate the general anesthesia and pneumoperitoneum.

Our work had certain limitations. First, the sample size was small (*n* = 447), especially that of the laparoscopic surgery subgroup (*n* = 39), making comparisons between open and laparoscopic surgery groups relatively difficult. Even though, we confirmed that laparoscopic surgery is as safe as open surgery for elderly inguinal–hernia patients, which was similar to those of previous studies [5, 14–18]. Moreover, in order to reduce potential selection bias, 1:1 propensity score matching was performed between open and laparoscopic surgery groups based on patients’ demographics and comorbidities, which makes the results convincing. Meanwhile, all postoperative outcomes were also comparable between the two groups under the general anesthesia subgroup, which further makes the results convincing. In addition, the small sample size of laparoscopic surgery subgroup rendered comparisons between traditional care and SMC less credible. However, most of patients (82.1%, 32/39) underwent laparoscopic surgery by highly surgeons with experienced laparoscopic skills in recent 2 years, and the results were consistent with real-world clinical practice. Second, any retrospective study has the inherent limitation that unobserved confounding variables may be in play. A large, prospective multicenter study is needed for the generalization of the findings. Finally, we calculated only the overall rates of postoperative complications during hospitalization, not the short-term (within 30 days of discharge) or long-term (within 6 or 12 months of discharge) rates. However, most postoperative complications develop during hospitalization.

## Conclusions

We confirmed that laparoscopic, inguinal hernia repair was safe for elderly patients, especially those with multiple comorbidities under SMC.

## Data Availability

Data are available on request to the corresponding author.
